# Compression of common peroneal nerve caused by an extraneural ganglion cyst mimicking intermittent claudication

**DOI:** 10.1186/1749-7221-8-5

**Published:** 2013-05-30

**Authors:** Raif Ozden, Vedat Uruc, Aydıner Kalacı, Yunus Dogramacı

**Affiliations:** 1 Department of Orthopaedics and Traumatology, Faculty of Medicine, Mustafa Kemal University, Antakya, Hatay, Turkey

**Keywords:** Peroneal nerve, Ganglion cyst, Tibiofibular joint

## Abstract

Peripheral neuropathies caused by ganglion cysts are rare. They seldom cause serious complications especially in the lower extremities. The case was a 51-year-old woman referred by her physician to the vascular surgeon with diagnosis including intermittent (vascular) claudication and deep venous thrombosis. Primarily vascular surgeon performed a doppler ultrasound of the lower extremity and calculation of the ankle-brachial index. There were no abnormal pathological findings. Careful physical examination revealed soft swelling and tenderness around the fibular head and neck. Weakness was observed in foot eversion and dorsiflexion. There was pain and tingling in the distribution of the peroneal nerve. and referring the patient to orthopedic surgeon owing to concern for a potential compressive lesion at the right proximal tibiofibular region. Electromyogram studies and physical examination confirmed a diagnosis of compression neuropathy of common peroneal nerve. Magnetic resonance imaging revealed a fluid-filled, lobulated mass indicating a ganglion cyst. One months after decompression, the patient had no complaint. Fast diagnosis and immediate management are essential to regain best possible recovery.

## Introduction

Leg pain, loss of ankle dorsiflexion and sensory loss,neurogenic claudication most commonly produced by degenerative disc disease of the lumbar spine. On the other hand, isolated peroneal nerve compression can mimic lumbar disc disease. Differentiation of symptoms of vascular claudication from symptoms of neurogenic claudication is important. Ganglion cyst is the most frequent tumours of the upper extremity. Despite their high incidence, ganglion cyst rarely result in peripheral nerve compression [1]. Compression neuropathies of the lower extremity are much less common and comprise only a minority of cases have been described [2-9]. The peak incidence has been seen at the fourth decade of life and it is rare in children [2,8]. Neurologic symptoms and pain are typical manifestations. We describe a case with a ganglion cyst as a rare cause of peroneal neuropathy and mimicking intermittent claudication treated surgically.

## Case presentation

**Figure 1:**
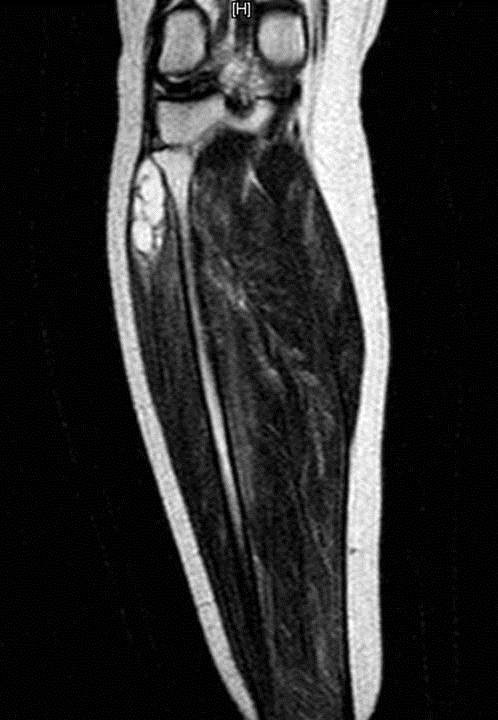
Characteristic magnetic resonance imaging findings of ganglion cyst with high signal intensity on the T2 sequence images.

**Figure 2:**
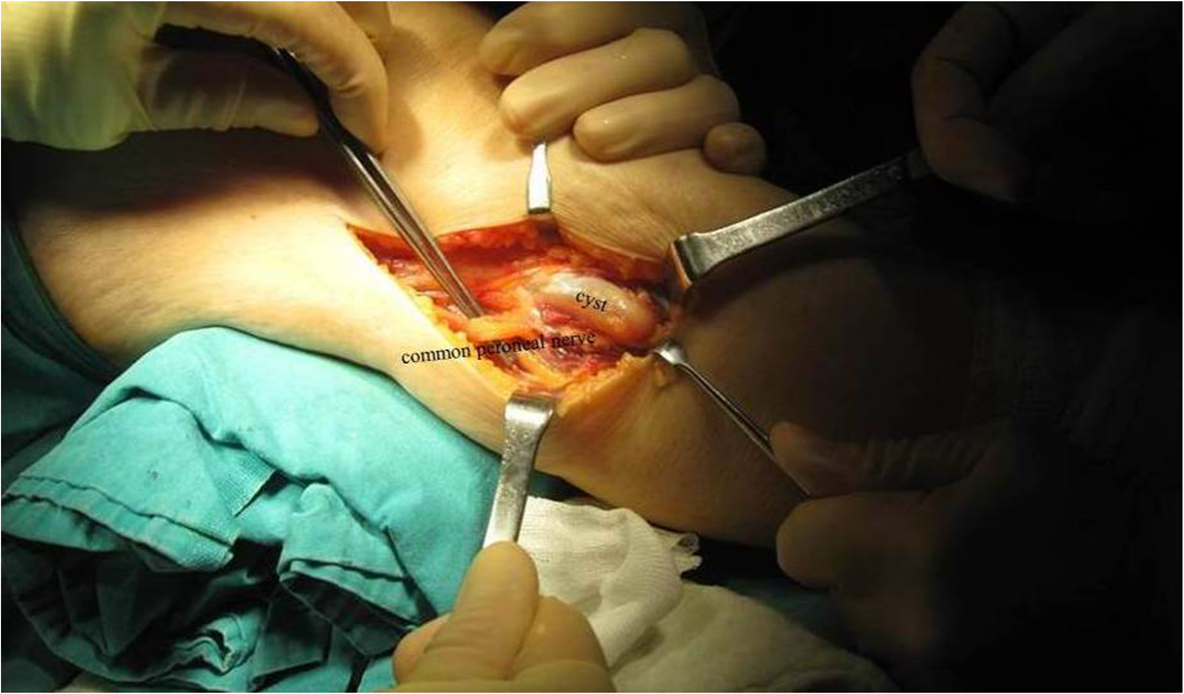
Intraoperative photograph of the lesion as seen associated with the peroneal nerve.

A 51-year-old female patient presented to her physician with a six-month history of intermittent claudication. She developed aching, cramping, pain and weakness of her right calf. She has radiating pain and hypoesthesia, while motor weakness was less prominent. She was referred to a vascular surgeon. After examination of the case there was nothing that concerns vascular surgery. Tenderness in the area of the right fibular head with gradual development of swelling in the same area was determined. Therefore,case was referred to orthopedic surgeon. A comprehensive physical examination revealed soft tissue swelling in the region around the fibular head and neck. There was slightly weakness in foot eversion and dorsiflexion, especially of the first toe. Inversion was normal. Electromyogram studies of the common peroneal nerve demonstrated significant neuropathic abnormalities. Subsequent magnetic resonance imaging demonstrated a lobulated, multilocular,cystic-appearing mass around the proximal fibular area. It was measured approximately 3 cm × 2 cm × 2 cm (Figure 1). The lesion was located anterior to the lateral aspect of the fibular neck, with the common peroneal nerve compressed against the posterior aspect of the cyst. It extended along the nerve toward its bifurcation. Using a lateral approach, the common peroneal nerve was recognized and traced to its bifurcation (Figure 2). The mass was followed down to its stalk and removed completely. The peroneal nerve was recognized as intact. All nerve branches were preserved under loupe magnification. The surgical material was diagnosed as ganglion cyst by histopathological examination. After one month of the surgery, the patient had no complaints of pain and the claudication also recovered completely.

## Discussion

Peripheral nerve lesions owing to ganglionic cysts are infrequent findings. The pathogenesis of these cysts has been the subject of controversy. However, evidence has demonstrated that they are of articular origin [10,11]. Ganglionic cysts compressing the peroneal nerve may be extraneural or intraneural [12]. Most of the ganglionic cysts defined in the literature causing peroneal nerve compression were intraneural type. Compression of the peroneal nerve was owing to an extraneural cyst as in our case is a condition rarely encountered and history of knee trauma is a common findingd [13,14]. There was knee trauma history in our case. The most frequent symptoms of this condition are weakness in the tibialis anterior, peroneus longus and brevis muscles, and pain radiating to the lateral malleolus [15-17]. Swelling of the proximal tibio-fibular joint are less common. Our case presented with radiating pain and hypoesthesia, while motor weakness was less prominent. The other complaints were swelling and localized pain in the region around the fibular head and neck. The differential diagnosis should include root compression, a nerve compression near the tendinous arch of the peroneal longus muscle, a nerve-sheath tumor, the osteocartilaginous exostosis at the proximal lower leg [18-20] and intermittent claudication as in our case. Plain radiographs have little importance in the diagnosis of ganglion cyst, but may be beneficial in eliminating a bony anomaly or fracture of the proximal part of fibula. Furthermore it may be useful in excluding degenerative disc disease of the lumbar spine. Magnetic resonance imaging is more useful in terms of the diagnosis. It may be difficult to differentiate a ganglion cyst from nerve sheath tumors and also solid masses on magnetic resonance imaging. Ultrasonography may be effective in showing the cystic nature of the mass and in differentiating it from solid tumors [21]. Compression of the fibular nerve by an extraneural ganglion is an infrequent and often misleading condition. If the patient has intermittent claudication especially with paresthesia, weakness in foot eversion and dorsiflexion, ganglion cyst should be considered in the differential diagnosis. A combination of magnetic resonance imaging and ultrasonography is helpful for correct diagnosis of the disorder, and it should be treated by microsurgical technique when possible.

## Conclusion

When a patient presenting with intermittent claudication, compression neuropathy of the peroneal nerve secondary to a ganglion cyst should be kept in mind in aspect of the differential diagnosis. After a complete history and physical examination, electromyogram and magnetic resonance imaging should be performed in terms of the differential diagnosis of a ganglion cyst. Careful preoperative evaluation, and early surgical excision by microsurgical technique in the management of the ganglion cyst should be recommended.

## Consent

Written informed consent was obtained from the patient for publication of this case report and accompanying images.

## Competing interests

We declare that we have no competing interests.

## Authors’ contributions

AK is the chief author who deals the patient clinically, BCG draws SKM attention for this case, RO and VU help AK in every aspect and YD take care of this patient preoperatively. All authors have read and approved the final manuscript.

## Authors’ information

Corresponding Author: Raif Ozden M.D. Assistant Professor. Mustafa Kemal University Faculty of Medicine, Department of Orthopaedics and Traumatology, Antakya, Hatay, Turkey.

Vedat Uruc M.D. Assistant Professor. Kemal University Faculty of Medicine, Department of Orthopaedics and Traumatology, Antakya, Hatay, Turkey.

Aydıner Kalacı M.D. Associate Professor. Mustafa Kemal University Faculty of Medicine, Department of Orthopaedics and Traumatology, Antakya, Hatay, Turkey.

Yunus Dogramacı M.D. Associate Professor. Mustafa Kemal University Faculty of Medicine, Department of Orthopaedics and Traumatology, Antakya, Hatay, Turkey.
